# Barley-Based Cereals Enhance Metabolic Health and Satiety in Overweight Korean Adults: A Randomized Trial

**DOI:** 10.3390/nu17172801

**Published:** 2025-08-28

**Authors:** Ingyeong Kang, Hyunsook Jang, Minchul Gim, Sang Eun Bae, Yu Jin Lee, Chai Sun Leem, Yoo Kyoung Park

**Affiliations:** 1 Department of Medical Nutrition, Graduate School of East-West Medical Science, Kyung Hee University, Yongin-si 17104, Republic of Korea; ingyeong22@khu.ac.kr (I.K.); 2025510044@khu.ac.kr (H.J.); 2 Lotte R&D Center, Seoul 07573, Republic of Korea; minchul.gim@lotte.net (M.G.); sangeun.bae@lotte.net (S.E.B.); 3 National Korean Medical Clinic, Seoul 06179, Republic of Korea; leeeyujin@gmail.com (Y.J.L.); altermedi@hanmail.net (C.S.L.)

**Keywords:** barley, dietary fiber, meal replacement, satiety, glycemic control, insulin resistance, body composition, GLP-1

## Abstract

**Background/Objectives:** Recent shifts in dietary patterns have led to reduced fiber consumption, corresponding with increasing rates of obesity and metabolic disorders. Barley-based cereals with high fiber content, particularly β-glucan, may provide superior metabolic and satiety benefits compared to corn-based alternatives. This study investigated whether barley-based cereals provide superior metabolic and satiety benefits compared to corn-based alternatives in overweight Korean adults. **Methods:** After selecting the most optimal cereal in the phase 1 study (acute postprandial research), a 6-week randomized controlled trial (RCT) was conducted in phase 2. In the phase 2 trial, overweight adults (*n* = 30; mean age of 43 ± 10.89 years; 36.7% female) were randomly assigned to consume either barley (*n* = 15) or corn cereal (*n* = 15) daily for 6 weeks. Participants consumed approximately 50 g of available carbohydrates (either barley or corn cereal) in 190 mL milk. Outcome measures included anthropometric parameters, fasting blood glucose, homeostasis model assessment of insulin resistance (HOMA-IR), postprandial glucose, subjective satiety, and gut health. **Results:** After 6 weeks, between-group comparisons revealed significant differences favoring the barley group in body weight (barley: −0.33 kg vs. corn: +0.85 kg; difference: −1.18 kg, *p* = 0.027), BMI (barley: −0.14 kg/m^2^ vs. corn: +0.03 kg/m^2^; difference: −0.17 kg/m^2^, *p* = 0.014), and glycated albumin (barley: −0.78% vs. corn: +0.09%; difference: −0.87%, *p* = 0.032). Within-group analyses showed that the barley group exhibited significant reductions in percent body fat (−1.03%, *p* = 0.004), waist circumference (−3.64 cm, *p* = 0.003), waist-to-hip ratio (−0.02, *p* = 0.012), glycated albumin (−0.78%, *p* = 0.029), and LDL cholesterol (−10.57 mg/dL, *p* = 0.033). Conversely, the corn group showed significant increases in body weight (+0.85 kg, *p* = 0.026) and percent body fat (+0.84%, *p* = 0.020), with a significant decrease in HDL cholesterol (−2.84 mg/dL, *p* = 0.020). **Conclusions:** Barley-based cereals offer significant metabolic and satiety benefits for overweight adults compared to corn-based alternatives. These findings suggest that barley-based cereals may be an effective dietary intervention for managing obesity and metabolic disorders.

## 1. Introduction

The global obesity epidemic has emerged as one of the most pressing public health challenges of the 21st century [1], with Korea experiencing a particularly rapid transformation in metabolic health outcomes [2]. Recent data from the Korea National Health and Nutrition Examination Survey (KNHANES) (2014–2023) reveal that, while the prevalence of hypertension and diabetes among adults aged 19 years and older has remained relatively stable, the rates of obesity and hypercholesterolemia have steadily increased [3], emphasizing the urgent need for effective dietary strategies [4].

Modern diets increasingly resemble swift currents, drawing individuals from the slow simmer of traditional cooking toward the instant gratification of convenience foods. This shift, driven by the accelerating pace of daily life, has fueled a growing demand for ready-to-eat options that fit seamlessly into busy schedules. This dietary transformation runs parallel to the increase in dining out, which has risen from 32.1% in 2007 to 41.0% in 2017, as reported in the KNHANES data [5]. Such changes reflect a broader societal pivot where time scarcity prompts individuals toward solutions that promise efficiency over effort, frequently at the expense of nutritional quality. The coronavirus disease 2019 pandemic has served as a catalyst for this trend, with Koreans reducing their restaurant dining substantially while turning to home meal replacements, demonstrating how external circumstances can accelerate shifts in dietary habits [6,7].

This transformation has particularly impacted dietary fiber intake, a critical nutrient for metabolic health. Data from the KNHANES conducted between 2010 and 2012 showed a notable downward trend [8], while similar analyses from 1998 to 2009 confirm that the average dietary fiber intake among Korean adults has fallen substantially below the recommended levels [9]. This decline is particularly evident in the reduced consumption of traditional fiber-rich whole grains, including barley and mixed grains, which were once staples of the Korean diet [10,11]. Adequate dietary fiber intake reduces the risk of obesity, type 2 diabetes, and cardiovascular disease through multiple mechanisms: delaying gastric emptying, reducing postprandial glucose spikes, enhancing insulin sensitivity, and promoting satiety [12]. Among fiber-rich whole grains, barley has demonstrated particular promise due to its high β-glucan content and metabolic benefits [13,14].

The growing need for effective meal replacement for weight management among overweight adults has led to a sharper focus on practical dietary solutions [15]. Commercial breakfast cereals tend to prioritize taste and convenience in their formulations [16]. While barley has been part of traditional Korean diets and contains substantial β-glucan [10,11], it is not commonly used in cereal products. To address this gap, we first compared acute postprandial responses to cereals made from different grains to select the optimal grain type, then conducted a longer intervention to evaluate its metabolic benefits.

Given the growing prevalence of obesity and metabolic disorders in Korea, the objective of the current study was to comprehensively evaluate the efficacy of barley-based cereal as a meal replacement in improving glycemic control, satiety, and overall metabolic health compared to corn-based alternatives in overweight Korean adults.

## 2. Materials and Methods

### 2.1. Recruitment and Population

Thirty participants were recruited from the general public through posters and advertisements placed around hospitals and local communities in Seoul, Korea.

Inclusion criteria were as follows: (1) adults with body mass index (BMI) between 23 and 29.9 kg/m^2^ [4] or those with InBody-measured body fat percentage of ≥25% for males and ≥30% for females; (2) individuals who consume breakfast at least 5 times per week; (3) individuals capable of consuming cereal with milk 6 days per week (excluding days when they cannot eat proper meals); and (4) those who voluntarily provided written informed consent. Exclusion criteria were as follows: individuals who (1) had experienced substantial weight gain or loss (>3 kg) in the previous 6 months; (2) were diagnosed with diabetes; (3) were allergic to or could not consume cereals or milk; (4) had food allergies or restricted diets; and (5) were pregnant women, nursing mothers, or women planning pregnancy during the study period. The study was conducted in accordance with the Declaration of Helsinki and approved by the Institutional Review Board of Kyung Hee University (KHGIRB-24-368). This trial was registered at CRIS (KCT0010461, 8 May 2025) and reported in accordance with CONSORT 2025 guidelines (Supplementary Materials S1).

According to previous studies on cereal consumption and metabolic responses, a minimum of 12 participants per group is required to detect meaningful differences in the postprandial glucose response. Considering a potential dropout rate of 20%, we decided on a total sample size of 30 participants (*n* = 15/group) [17,18].

**Figure 1:**
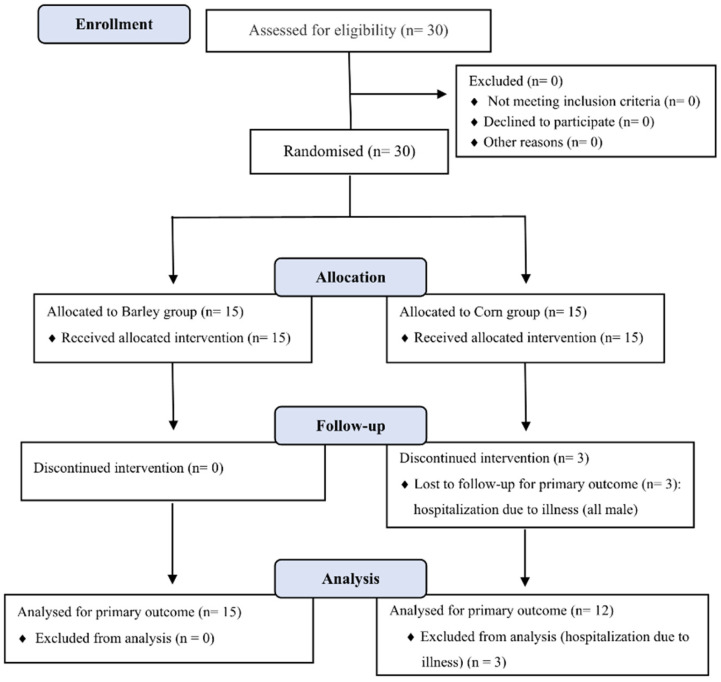
CONSORT flow diagram of study participants. Thirty participants were randomized to barley (*n* = 15) or corn (*n* = 15) groups. Three participants in the corn group withdrew due to unrelated hospitalization. Final analysis: barley (*n* = 15), corn (*n* = 12).

An acute postprandial assessment (Phase 1) was conducted with 15 participants to select the optimal cereals. In the phase 2 study, participants were randomly assigned to either the barley group (*n* = 15) or corn group (*n* = 15) using block randomization consisting of block sizes of four, including interventions and control groups at a 1:1 ratio; this was prepared in advance from a random number sequence generated using SAS 9.4 by a researcher who was not involved in participant registration. The participant flow diagram of the study is shown in Figure 1.

### 2.2. Study Design (Phase 1)

The phase 1 study examined the metabolic and satiety effects of different cereal grains. Based on our preliminary analysis, we selected barley, wheat, and corn for comparison. The participants consumed their assigned cereals in flake form after an overnight fast of 10–12 h. A standardized portion containing approximately 50 g of available carbohydrates was served with 190 mL of sterilized milk. Table 1 shows the nutritional components of the three cereal types evaluated in phase 1. For phase 1, we used whole grains that underwent only mechanical pressing/flaking—the minimal processing required to make raw grains edible. These minimally processed grain flakes contained no additives whatsoever, allowing us to assess the acute glycemic responses of the grains themselves. Blood samples were collected via finger prick at baseline (0 min) and at 30, 60, 90, and 120 min postprandially to measure capillary blood glucose levels (see Section 2.5 for detailed methodology). Subjective satiety was simultaneously assessed using a validated VAS at identical time points.

**Figure 2:**
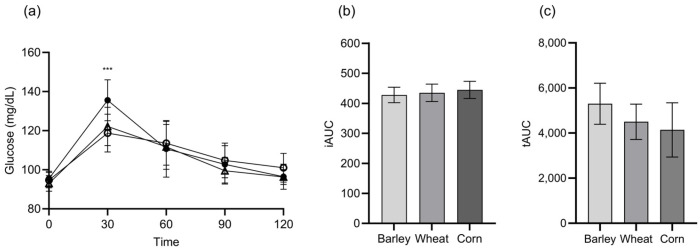
(**a**) Postprandial glucose responses over 120 min, (**b**) incremental area under the curve (iAUC) for glucose response, and (**c**) total area under the curve (tAUC) for subjective satiety ratings after consumption of barley, wheat, and corn flakes. ○: Barley; △: Wheat; ●: Corn. Data are presented as means ± standard deviation. Statistical analysis by a one-way ANOVA. *** *p* < 0.001 vs. barley group at 30 min. Abbreviations: iAUC, incremental area under the curve; tAUC, total area under the curve.

As shown in Figure 2, the postprandial glucose response curves revealed that corn flakes produced a significantly higher glucose peak at 30 min than barley and wheat flakes, whereas barley flakes resulted in the most gradual increase and decrease in blood glucose levels throughout the 120-min period. Glucose iAUC analysis (Figure 2b) showed no significant differences between groups (barley: 427.9 ± 27.8; wheat: 433.8 ± 31.2; corn: 444.9 ± 30.4 mg·min/dL; *p* > 0.05). Subjective satiety ratings (Figure 2c) indicated that barley flakes produced the highest feelings of satisfaction among the participants, followed by wheat and corn flakes, although these differences were statistically nonsignificant. Barley flakes produced more stable postprandial glucose patterns than corn flakes, with significantly lower glucose peaks at 30 min (118.73 ± 9.40 mg/dL for barley vs. 135.53 ± 10.12 mg/dL for corn; *p* < 0.001). Based on these combined results, barley was selected for the phase 2 intervention study.

### 2.3. Study Design (Phase 2)

Based on the phase 1 analysis, barley, which resulted in the most favorable postprandial glucose response, was selected as the experimental group. In contrast, corn, which led to the least favorable response, was chosen as the control group. Participants were provided with cereals in pre-packaged portions, with instructions to consume one serving each morning with 190 mL of sterilized milk for 6 weeks. Each serving consisted of approximately 60 g of cereal, providing approximately 50 g of available carbohydrates. The cereals were provided in identical, unmarked packaging to ensure participant blinding to the type of cereal they were consuming. However, due to differences in appearance and taste between barley and corn cereals, complete blinding could not be guaranteed.

Participants were instructed to maintain their usual physical activity and dietary habits throughout the study, except for the addition of the test cereals. A comprehensive questionnaire was administered at baseline to assess potential confounding factors such as supplement use and consumption of foods that might influence the study outcomes. Compliance was monitored by collecting and counting the number of cereal packages remaining at the end of the study period, with additional weekly remote counseling sessions being conducted to verify the quantity of cereal consumption.

At baseline (week 0) and endpoint (week 6), the following clinical assessments were conducted: fasting blood samples for glucose, insulin (for calculation of HOMA-IR), lipid profile, inflammatory markers, and glucagon-like peptide 1 (GLP-1) levels; anthropometric measurements (body weight, BMI, percent body fat, waist, and hip circumference); and completion of validated questionnaires regarding satiety and gastrointestinal comfort using VAS.

### 2.4. Products

Two customized cereal products were formulated and manufactured for this study: barley- and corn-based cereals. These experimental products were developed in collaboration to ensure a standardized composition while differing primarily in their grain base. Table 2 summarizes the complete nutritional compositions of both products. The barley-based experimental cereal contained 197.82 kcal per serving, with 53.68 g of carbohydrates, 0.47 g of sugars, 7.83 g of dietary fiber, 4.77 g of protein, and 0.85 g of fat. The corn-based experimental cereal provided 211.11 kcal per serving, with 53.35 g of carbohydrates, 0.69 g of sugars, 4.73 g of dietary fiber, 4.3 g of protein, and 2.14 g of fat. The most notable difference between the two formulations was the dietary fiber content, with barley cereals containing approximately 65.5% more fiber than corn cereals. Both cereals were produced using identical processing techniques to minimize confounding variables beyond the primary grain composition. Cereals were served with 190 mL of sterilized milk, to which 107 kcal, 6 g of carbohydrates (all as sugars), 6 g of protein, and 6.5 g of fat were added. As specified by the manufacturer, barley cereals underwent additional processing compared to corn cereals owing to their smaller and harder grains. Barley requires a pretreatment process to soften its texture and enable aggregation into pellets. Corn production typically involves steaming, pressing (shaping), syrup coating, and drying. Conversely, barley cereal production includes pretreatment, steaming, pelletization (aggregation), pressing, syrup coating, and drying.

### 2.5. Anthropometric and Clinical Measurements

Height was self-reported by participants at baseline. Body weight and composition, including percent body fat, were assessed using bioelectrical impedance analysis (InBody 270, InBody Co., Seoul, Republic of Korea) after a 10–12 h overnight fast, with participants barefoot and wearing light clothing.

Waist circumference was measured at the midpoint between the lowest rib and the iliac crest, and hip circumference was measured at the widest point of the buttocks, using a non-stretchable tape measure. All measurements were taken twice by a trained researcher and averaged.

Blood pressure was measured by a trained and qualified researcher using an automated oscillometer (OMRON HEM-7120, Omron Healthcare, Kyoto, Japan). After participants had rested in a seated position for at least 5 min, two measurements were taken at 1-min intervals, and the average was recorded.

### 2.6. Blood Analysis

Participants arrived at the laboratory after a 10–12 h overnight fast. Blood samples were collected via venipuncture for baseline measurements. For the acute glucose response test in phase 1, finger-prick blood samples were collected at 0, 30, 60, 90, and 120 min post-consumption using an automatic lancing device by trained research personnel. Blood glucose levels were measured immediately using a self-monitoring blood glucose meter (ACCU-CHEK Performa; Roche Diagnostics, Seoul, Republic of Korea). All measurements followed standardized procedures, with re-testing performed when error messages occurred.

Venous blood samples were collected at baseline and after the 6-week intervention period to assess metabolic markers in phase 2 of the study. Blood samples were centrifuged at 3000 rpm for 15 min to separate the serum and plasma, which were stored at −80 °C until analysis. Fasting glucose, insulin (for calculation of HOMA-IR), glycated albumin [19], *C*-reactive protein, lipid profile (total cholesterol, low-density lipoprotein [LDL] cholesterol, high-density lipoprotein [HDL] cholesterol, and triglycerides), and GLP-1 levels were analyzed [20,21]. Insulin resistance was assessed using homeostasis model assessment of insulin resistance (HOMA-IR), which was calculated using the following formula: HOMA-IR = fasting insulin (μU/mL) × fasting glucose (mg/dL)/405 [22].

### 2.7. Satiety (VAS)

Subjective appetite was assessed using a 100 mm VAS administered at baseline and after 6 weeks of cereal consumption for both the barley (*n* = 15) and corn (*n* = 12) groups. The appetite assessment consisted of four key dimensions: hunger level (from “not hungry at all: 0 mm” to “intolerably hungry: 100 mm”), fullness level (from “not full at all: 0 mm” to “excessively full: 100 mm”), satisfaction level (from “completely empty: 0 mm” to “cannot eat anything: 100 mm”), and desire to eat (from “cannot eat anything: 0 mm” to “capable of eating a lot: 100 mm”). Higher scores for hunger indicated more severe hunger, whereas higher scores for fullness and satisfaction indicated a greater intensity of these sensations [23].

### 2.8. Gut Quotient (GQ)

The Korean version of the GQ measurement scale was used to evaluate intestinal health [24]. This culturally adapted and validated assessment tool comprises 17 distinct items that comprehensively evaluate various aspects of gastrointestinal function, including digestive comfort, bowel movement patterns, and abdominal sensations. Responses to each item were recorded on a 5-point Likert scale, with the cumulative scoring system designed to ensure that higher total scores reflect better overall gut health and function.

A comparative analysis of changes in the GQ scores was performed to determine the differential impact of barley and corn cereals on the gastrointestinal comfort and function of participants. Particular attention was directed toward monitoring potential digestive adaptations or discomfort that may accompany increased dietary fiber consumption, especially in the barley group, such as temporary bloating, changes in flatulence, or alterations in normal bowel patterns during the adaptation period.

### 2.9. Statistical Analysis

Data are presented as mean ± standard deviation. Statistical analyses were performed using IBM SPSS Statistics for Windows, Version 28.0 (Armonk, NY, USA). The Shapiro–Wilk test was used to assess normality at a 5% significance level.

For the acute postprandial assessment in phase 1, differences between the groups were evaluated using the independent *t*-test when normality was confirmed (*p* > 0.05, Shapiro–Wilk test) and the Mann–Whitney U test when the distribution was non-normal (*p* < 0.05, Shapiro–Wilk test). Statistical significance is indicated by asterisks (* *p* < 0.05, ** *p* < 0.01, *** *p* < 0.001) in all tables and figures. Incremental area under the curve (iAUC) for the glucose response and total area under the curve (tAUC) for subjective satiety ratings (hunger, fullness, satisfaction, and desire to eat) were calculated using the trapezoidal method in Microsoft Excel and GraphPad Prism version 10.0 (GraphPad Software, Inc., La Jolla, CA, USA). For the phase 2 study, similar parametric or non-parametric tests were applied to evaluate changes in outcome measures from baseline to study completion (Δ6 weeks-0 weeks) based on the normality of data distribution. Primary outcomes included anthropometric measurements (body weight, BMI, percent body fat, waist and hip circumference, and waist-to-hip ratio), blood measurements (fasting glucose, glycated albumin, liver enzymes, *C*-reactive protein [CRP], and lipid profiles), GLP-1 levels, and HOMA-IR for insulin resistance. The level of statistical significance was set at *p* < 0.05 in a two-sided test.

## 3. Results

### 3.1. General Participant Characteristics

Thirty participants were recruited and randomly allocated to the barley (*n* = 15) and corn (*n* = 15) groups. Table 3 presents the baseline characteristics of both groups. No significant differences observed in age (45 ± 12.61 vs. 42 ± 9.23 years), BMI (27.1 ± 4.0 vs. 26.5 ± 2.7 kg/m^2^), body weight (81.1 ± 16.6 vs. 74.0 ± 12.4 kg), or other anthropometric parameters.

### 3.2. Changes in Anthropometric and Metabolic Parameters

Table 4 presents changes in anthropometric measures after cereal consumption. The barley group exhibited significant reductions in percent body fat (31.88 ± 7.91% to 30.85 ± 7.91%; *p* = 0.004) and waist circumference (89.27 ± 8.42 cm to 85.63 ± 10.38 cm; *p* = 0.003). Additionally, the waist-to-hip ratio was significantly reduced in the barley group (0.87 ± 0.06 to 0.85 ± 0.08; *p* = 0.012). Conversely, the corn group showed a significant increase in percent body fat (30.44 ± 5.31% to 31.28 ± 5.00%; *p* = 0.020) and body weight (76.43 ± 14.33 kg to 77.28 ± 11.43 kg; *p* = 0.026). Between-group comparisons revealed significant differences, favoring the barley group in terms of body weight (difference: −1.18 kg, *p* = 0.027) and BMI (difference: −0.17 kg/m^2^, *p* = 0.014). Neither group showed significant changes in blood pressure or pulse rate.

The changes in metabolic parameters are detailed in Table 5. Consumption of barley cereal significantly reduced glycated albumin levels (12.61 ± 0.66% to 11.83 ± 0.69%; *p* = 0.029). The barley group also showed a significant reduction in LDL cholesterol levels (117.85 ± 30.88 mg/dL to 107.28 ± 31.04 mg/dL; *p* = 0.033). Conversely, the corn group exhibited a significant decrease in HDL cholesterol (49.17 ± 12.33 mg/dL to 46.33 ± 11.41 mg/dL; *p* = 0.023). No significant changes were observed in fasting glucose, CRP, total cholesterol, or triglyceride levels in either group.

### 3.3. Changes in Glucose Metabolism and Postprandial Response

**Figure 3:**
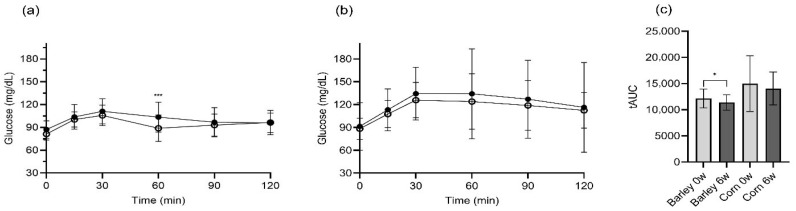
Glycemic responses and tAUC 120 min after barley or corn cereal consumption. (**a**) Postprandial glucose response in the barley group. (**b**) Postprandial glucose response in the corn group. (**c**) Total area under the curve (tAUC) comparison between baseline and 6-week measurements for both groups. ●: Baseline; ○: 6 weeks. Data are presented as means ± standard deviation. Statistical analysis was performed using Wilcoxon signed-rank test for within-group comparisons. * *p* < 0.05, *** *p* < 0.001. Abbreviation: tAUC, total area under the curve.

As shown in Figure 3, glucose responses differed between barley and corn cereal consumption. The barley group demonstrated a significantly lower glucose response 60 min post-intervention (Figure 3a). In contrast, the corn group showed minimal changes in glucose response patterns between the baseline and post-intervention measurements (Figure 3b). According to the area under the curve (AUC) analysis, the barley group exhibited a statistically significant reduction in glucose AUC values after the intervention, whereas the corn group showed no significant change over time (Figure 3c). Individual participant responses demonstrated marked differences between groups (Supplementary Figure S1). In the barley group, 13 of 15 participants (86.7%) showed reduced glucose tAUC after 6 weeks, while in the corn group, only 5 of 12 participants (41.7%) showed improvements, with high individual variability contributing to the lack of statistical significance.

### 3.4. Changes in GLP-1 Levels

After the 6-week intervention, the barley group showed a significant increase in GLP-1 levels at 30 min postprandially (from 34.61 ± 23.09 pmol/L to 44.48 ± 32.48 pmol/L; *p* = 0.044). No significant changes in GLP-1 levels were observed in the corn group at any measured time point. The between-group difference at 30 min was statistically significant (*p* = 0.040), suggesting that barley consumption may enhance early postprandial GLP-1 secretion compared with corn consumption. Table 6 shows the GLP-1 values and changes over 120 min for both groups. The GLP-1 response curves (Supplementary Figure S2) demonstrate that barley consumption enhanced the early postprandial GLP-1 peak, with the maximum difference occurring at 30 min.

### 3.5. Changes in Fasting Insulin and Insulin Resistance After Barley and Corn Cereal Consumption

Table 7 shows the changes in fasting insulin and HOMA-IR values. The barley cereal group exhibited a significant improvements in both fasting insulin levels and insulin resistance. Fasting insulin decreased from baseline (10.6 ± 4.4 μU/mL) to post-intervention (8.3 ± 4.1 μU/mL; *p* = 0.012), accompanied by a significant reduction in HOMA-IR from 2.32 ± 1.03 to 1.47 ± 0.67 (*p* = 0.012). In contrast, the corn cereal group showed no significant changes in either parameter, with fasting insulin levels remaining stable (9.0 ± 2.5 to 9.2 ± 3.6 μU/mL; *p* = 0.889) and HOMA-IR showing only a nonsignificant reduction from baseline (2.28 ± 1.62) to post-intervention (1.63 ± 1.18; *p* = 0.532). Between-group comparisons revealed that barley cereal consumption resulted in significantly greater improvement than corn cereal consumption in both fasting insulin (*p* = 0.038) and insulin resistance (difference: −0.55, *p* = 0.000).

### 3.6. GQ

Following 6 weeks of cereal consumption, the barley cereal group showed a slight decrease in GQ values from baseline (86.36 ± 22.29) to post-intervention (84.81 ± 8.44), although this difference failed to reach statistical significance (*p* = 0.061). Likewise, the corn cereal group demonstrated a decreasing trend in GQ values from baseline (85.39 ± 11.98) to post-intervention (78.26 ± 12.42), although this difference did not reach statistical significance (*p* = 0.060). The difference in GQ scores between the two groups was statistically nonsignificant (*p* = 0.514). These findings suggested that both cereals exert similar effects on gut function.

### 3.7. tAUC of VAS

Table 8 presents the comparison of tAUC values for VAS measurements between the barley and corn groups. Between-group comparisons revealed significant differences in fullness (*p* = 0.020) and satisfaction (*p* = 0.014) scores favoring the barley cereal group. Barley cereal consumption tended to increase fullness (baseline: 5475.00 ± 1892.45; post: 6300.00 ± 1789.34) and satisfaction (baseline: 4725.00 ± 1678.90; post: 5400.00 ± 1456.78) scores, whereas corn cereal consumption showed decreasing trends in both measures. Although statistically nonsignificant, the barley group demonstrated a greater reduction in hunger and desire to eat than the corn group.

## 4. Discussion

The present study provides evidence supporting the metabolic and satiety benefits of barley-based cereals compared with corn-based alternatives in overweight Korean adults. Our findings reveal considerable improvements in multiple health parameters following 6 weeks of barley cereal consumption, including positive changes in body composition, glycemic control, insulin resistance, and subjective satiety measures.

Participants who consumed barley cereal experienced notable reductions in percent body fat and waist circumference, suggesting favorable changes in body composition. These improvements align with those reported previously, which revealed the beneficial effects of dietary fiber on body weight and fat distribution [25]. The marked reduction in the waist-to-hip ratio observed in the barley group is particularly noteworthy, given that central adiposity is a stronger predictor of cardiometabolic risk than overall body weight [26]. In contrast, the corn group showed a notable increase in percent body fat and body weight, highlighting the differential effects of these two cereal types despite their similar caloric content.

The substantial reduction in glycated albumin levels observed in the barley group indicated improved glycemic control over the 6-week intervention period. Glycated albumin, which reflects the average glucose levels over approximately 2–3 weeks, has been recognized as a more useful marker for short-term glycemic control assessment than HbA1c [27]. This improvement was complemented by notably enhanced insulin sensitivity, as evidenced by a marked reduction in HOMA-IR values in the barley group. The substantial between-group differences in HOMA-IR changes (*p* = 0.000) underscore the superior effect of barley on insulin metabolism compared to that of corn. These findings are consistent with those of previous studies, demonstrating the insulin-sensitizing effects of dietary fiber, particularly β-glucan found in barley [28].

Furthermore, lipid profile changes differed between the groups, with the barley group demonstrating a substantial reduction in LDL cholesterol while maintaining HDL cholesterol levels. In contrast, the corn group showed a notable decrease in HDL cholesterol levels without beneficial changes in LDL cholesterol levels. These findings are consistent with previous research showing the cholesterol-lowering effects of barley β-glucan [29]. Maintaining HDL cholesterol levels in the barley group is particularly important, given the protective role of HDL against cardiovascular disease [30].

The substantial increase in GLP-1 levels 30 min postprandially in the barley group offers a potential mechanism for the observed satiety and metabolic benefits. GLP-1, an incretin hormone secreted by intestinal L cells in response to nutrient intake, plays a crucial role in glucose homeostasis by enhancing insulin secretion and suppressing glucagon release [31]. Furthermore, GLP-1 contributes to satiety regulation by delaying gastric emptying and acting on the central appetite regulatory centers [32]. The considerably higher GLP-1 response in the barley group than in the corn group suggests that the higher fiber content of barley may stimulate greater GLP-1 secretion, potentially explaining the enhanced satiety and improved glycemic control observed in this group [33].

The relatively large standard deviations observed in Figure 1 and Table 6 reflect the inherent biological variability in postprandial glucose and GLP-1 responses among our participants. This variability is consistent with similar studies in the literature and can be attributed to several factors, as follows: (1) individual differences in metabolic responses [34], (2) variation in baseline insulin sensitivity among overweight participants [35], (3) differences in gut microbiota composition that may influence fiber fermentation and GLP-1 secretion [33], and (4) genetic factors affecting carbohydrate metabolism [36]. Despite this variability, our statistical analysis confirmed significant differences between groups, reinforcing the robustness of our findings.

The marked improvements in fullness and satisfaction scores detected in the barley group aligned with the observed metabolic benefits and provided practical implications for dietary recommendations [23,37]. These findings suggest that barley-based cereals may help address the dual challenges of inadequate dietary fiber intake and the growing demand for convenient and satiating meal replacements among Korean adults. The consistent improvement across both objective and subjective measures strengthens our conclusion that barley cereals promote greater satiety despite having a lower caloric content than corn cereals. The present study has several methodological strengths. First, we employed a two-phase approach that integrated acute postprandial assessments with a 6-week intervention, allowing us to identify the optimal grain type before conducting longer-term evaluation. Second, we comprehensively evaluated multiple dimensions of metabolic health simultaneously, including glycemic control (glucose, glycated albumin, and HOMA-IR), insulin resistance, lipid profiles, body composition, and satiety markers, rather than focusing on isolated parameters. Third, we achieved high dietary compliance (>95%) through close monitoring, substantially enhancing the reliability of our findings. Fourth, the 100% barley-based cereals used in this study were optimized for sensory properties using innovative processing techniques while minimizing sugar content, allowing us to observe the pure effects of different grains. Finally, conducting this research during Korea’s nutritional transition period makes our findings particularly valuable for developing culturally appropriate dietary interventions.

Despite these promising findings, this study has several limitations. The limited sample size (barley group, *n* = 15; corn group, *n* = 12) and sex imbalance (19 males and 11 females) may restrict the generalizability of our results to broader populations. Additionally, the standardized nature of our test cereals, while ensuring controlled comparison, may not fully represent commercially available products, which vary in formulation and processing methods across brands. This should be considered when translating our findings to real-world dietary recommendations. Furthermore, the inability to achieve complete participant blinding due to inherent differences in cereal appearance and taste could have introduced expectation bias. The 6-week intervention period, while sufficient to observe significant changes, may not capture the full extent of potential metabolic adaptations that could occur with long-term consumption. Although participants were instructed to maintain their usual dietary habits, we could not completely rule out the influence of other dietary factors on the observed outcomes.

## 5. Conclusions

Barley-based cereals represent a practical dietary solution for managing obesity and metabolic disorders in the Korean population. The demonstrated ability to improve multiple metabolic parameters simultaneously through a simple breakfast substitution offers an accessible intervention strategy. The enhanced satiety despite lower caloric content provides a sustainable approach to weight management that may improve long-term adherence compared to restrictive diets.

This study validates the potential of traditional Korean grains, particularly barley, as functional foods in modern convenient formats. As Korea faces increasing rates of metabolic diseases alongside declining whole-grain consumption, incorporating barley into ready-to-eat products addresses both cultural dietary transitions and public health needs. These findings support the development of grain-based dietary guidelines that consider both metabolic benefits and practical implementation in contemporary lifestyles.

## Figures and Tables

**Table 1:** Nutritional composition of mechanically pressed whole-grain flakes (barley, wheat, and corn) used in the phase 1 acute response study.

	Milk	Barley		Wheat		Corn	
		Barley	Barley + Milk	Wheat	Wheat + Milk	Corn	Corn + Milk
Energy (kcal)	107	207.88 ^1^	314.88	220.48	327.48	237.45	344.45
Carbohydrate (g)	6	46.13	52.13	46.97	52.97	48.27	54.27
Sugars (g)	6	0.39	6.39	0.26	6.26	0.65	6.65
Dietary Fiber (g)	-	7.93	7.93	7.18	7.18	3.92	3.92
Protein (g)	6	5.75	11.75	8.48	14.48	5.22	11.22
Fat (g)	6.5	1.80	8.30	1.63	8.13	2.61	9.11
Saturated fat (g)	4	0.78	4.78	0.32	4.32	0.32	4.32
Trans fat (g)	0.5	0.02	0.52	0	0.5	0	0.5
Cholesterol (mg)	25	0	25	0	25	0	25
Sodium (mg)	70	2.05	72.05	1.30	71.30	3.92	73.92

^1^ Data are presented as means ± standard deviations.

**Table 2:** Nutritional components of barley and corn cereals per serving (60 g).

	Milk	Barley		Corn	
		Barley	Barley + Milk	Corn	Corn + Milk
Energy (kcal)	107	197.82	304.82	211.11	318.11
Carbohydrate (g)	6	53.68	59.68	53.35	59.35
Sugars (g)	6	0.47	6.47	0.69	6.69
Dietary Fiber (g)	-	7.83	7.83	4.73	4.73
Protein (g)	6	4.77	10.77	4.3	10.3
Fat (g)	6.5	0.85	7.35	2.14	8.64
Saturated fat (g)	4	0.22	4.22	0.27	4.27
Trans fat (g)	0.5	0	0.5	-	0.5
Cholesterol (mg)	25	0	25	-	25
Sodium (mg)	70	25.37	95.37	24.84	94.84

**Table 3:** Baseline characteristics of study participants.

	Total (*n* = 30)	Barley (*n* = 15)	Corn (*n* = 15)	*p*-Value
Age (years)	43 ± 10.89 ^1^	45 ± 12.61	42 ± 9.23	0.463
Male (*n*)	19	8	11	0.365
Female (*n*)	11	7	4	0.365
Height (cm)	169.4 ± 8.4	166.6 ± 9.2	172.2 ± 6.7	0.067
Body weight (kg)	77.6 ± 14.8	81.1 ± 16.6	74.0 ± 12.4	0.195
BMI ^2^ (kg/m^2^)	26.8 ± 3.4	27.1 ± 4.0	26.5 ± 2.7	0.634
PBF ^3^ (%)	30.7 ± 6.6	30.4 ± 5.3	30.9 ± 7.9	0.840

^1^ Data are presented as means ± standard deviations. *p*-values were calculated using independent *t*-test for continuous variables and Fisher’s exact test for categorical variables. ^2^ BMI, Body mass index. ^3^ PBF, Percent body fat.

**Table 4:** Effects of eating barley and corn cereal on anthropometric measures.

	Barley (*n* = 15)			Corn (*n* = 12)			
	Baseline	Post-6 Weeks	*p*-Value ^1^ Δ6 Weeks-0 Weeks	Baseline	Post-6 Weeks	*p*-Value ^1^ Δ6 Weeks-0 Weeks	*p*-Value ^1^
Body weight (kg)	74.02 ± 12.37 ^2^	73.69 ± 12.49	0.345	76.43 ± 14.33	77.28 ± 11.43	0.026 *	0.027 *
BMI ^3^ (kg/m^2^)	26.54 ± 2.73	26.40 ± 2.72	0.272	27.14 ± 3.97	27.17 ± 2.57	0.388	0.014 *
Percent body fat (%)	31.88 ± 7.91	30.85 ± 7.91	0.004 **	30.44 ± 5.31	31.28 ± 5.00	0.020 *	0.983
Systolic blood pressure (mmHg)	127.60 ± 12.08	126.60 ± 13.67	0.550	128.07 ± 15.26	129.42 ± 10.04	0.084	0.520
Diastolic blood pressure (mmHg)	87.00 ± 9.55	86.53 ± 7.10	0.977	88.53 ± 10.91	89.17 ± 8.78	0.141	0.917
Pulse rate (bpm)	71.53 ± 11.81	71.40 ± 12.99	0.861	68.87 ± 7.08	67.75 ± 6.81	0.610	0.212
Waist circumference (cm)	89.27 ± 8.42	85.63 ± 10.38	0.003 **	93.80 ± 10.60	89.50 ± 7.69	0.082	0.934
Hip circumference (cm)	102.07 ± 4.50	100.60 ± 4.07	0.061	103.87 ± 6.70	101.67 ± 5.40	0.509	0.281
Waist-to-hip ratio	0.87 ± 0.06	0.85 ± 0.08	0.012 *	0.90 ± 0.06	0.88 ± 0.05	0.122	0.379

^1^ Analyzed by Wilcoxon signed-rank test (within-group); Mann–Whitney test (between-group). * *p* < 0.05, ** *p* < 0.01. ^2^ Data are presented as means ± standard deviations. ^3^ BMI, body mass index.

**Table 5:** Effects of barley and corn cereal consumption on blood metabolic parameters.

	Barley (*n* = 15)			Corn (*n* = 12)			
	Baseline	Post 6 Weeks	*p*-Value ^1^ Δ6 Weeks-0 Weeks	Baseline	Post 6 Weeks	*p*-Value ^1^ Δ6 Weeks-0 Weeks	*p*-Value ^1^
Fasting glucose (mg/dL)	87.13 ± 11.44 ^2^	81.20 ± 7.88	0.132	91.47 ± 31.27	88.25 ± 14.14	0.308	0.693
Glycated albumin (%)	12.61 ± 0.66	11.83 ± 0.69	0.029 *	12.81 ± 1.03	12.90 ± 1.14	0.969	0.032 *
CRP ^3^ (mg/dL)	1.56 ± 2.99	0.76 ± 0.30	0.832	1.20 ± 1.33	1.11 ± 1.05	0.212	0.370
Total cholesterol (mg/dL)	195.75 ± 32.30	185.83 ± 23.17	0.158	191.71 ± 30.79	185.50 ± 33.47	0.158	0.709
Triglyceride (mg/dL)	125.67 ± 38.47	123.67 ± 34.47	0.842	173.64 ± 97.86	212.50 ± 142.97	0.071	0.237
LDL ^4^ cholesterol (mg/dL)	117.85 ± 30.88	107.28 ± 31.04	0.033 *	115.00 ± 32.08	118.83 ± 32.59	0.906	0.852
HDL ^5^ cholesterol (mg/dL)	53.33 ± 8.85	51.93 ± 11.55	0.281	49.17 ± 12.33	46.33 ± 11.41	0.023 *	0.123
Male (*n* = 8/8) ^6^	48.12 ± 5.54	47.00 ± 9.17	0.302	45.50 ± 11.46	41.62 ± 10.10	0.055	0.488
Female (*n* = 7/4) ^6^	59.29 ± 8.32	57.57 ± 12.00	0.812	56.50 ± 11.09	55.50 ± 9.95	0.875	1.000

^1^ Analyzed by Wilcoxon signed-rank test (within-group); Mann–Whitney test (between-group). * *p* < 0.05. ^2^ Data are presented as means ± standard deviation. ^3^ CRP, *C*-reactive protein. ^4^ LDL, low-density lipoprotein. ^5^ HDL, high-density lipoprotein. ^6^ *n* = Barley/Corn.

**Table 6:** GLP-1 values and changes over 120 min in the barley and corn groups.

	Barley (*n* = 15)			Corn (*n* = 12)			
	Baseline	Post 6 Weeks	*p*-Value ^1^ Δ6 Weeks-0 Weeks	Baseline	Post 6 Weeks	*p*-Value ^1^ Δ6 Weeks-0 Weeks	*p*-Value ^1^
GLP-1 ^3^ (pmol/L)							
0 min	25.53 ± 12.42 ^2^	30.15 ± 15.40	0.347	25.02 ± 15.32	31.44 ± 31.49	0.139	0.967
30 min	34.61 ± 23.09	44.48 ± 32.48	0.044 *	35.28 ± 33.00	38.56 ± 38.21	0.445	0.040 *
60 min	30.70 ± 16.30	37.90 ± 28.06	0.136	28.24 ± 24.45	30.85 ± 30.94	0.508	0.071
120 min	32.19 ± 20.86	33.16 ± 21.11	0.196	29.25 ± 25.88	30.66 ± 32.44	0.790	0.254

^1^ Analyzed by Wilcoxon signed-rank test (within-group); Mann–Whitney test (between-group). * *p* < 0.05. ^2^ Data are presented as means ± standard deviation. ^3^ GLP-1, glucagon-like peptide-1.

**Table 7:** Changes in fasting insulin and insulin resistance (HOMA-IR) before and after barley and corn cereal consumption.

	Barley (*n* = 15)			Corn (*n* = 12)			
	Baseline	Post 6 Weeks	*p*-Value ^1^ Δ6 Weeks-0 Weeks	Baseline	Post 6 Weeks	*p*-Value ^1^ Δ6 Weeks-0 Weeks	*p*-Value ^1^
Fasting insulin(μU/mL)	10.6 ± 4.4 ^2^	8.3 ± 4.1	0.012 *	9.0 ± 2.5	9.2 ± 3.6	0.889	0.038 *
HOMA-IR ^3^	2.32 ± 1.03	1.47 ± 0.67	0.012 *	2.28 ± 1.62	1.63 ± 1.18	0.532	0.000 ***

^1^ Analyzed by Wilcoxon signed-rank test (within-group); Mann–Whitney test (between-group). * *p* < 0.05, *** *p* < 0.001. ^2^ Data are presented as means ± standard deviation. ^3^ HOMA-IR, homeostatic model assessment of insulin resistance.

**Table 8:** Comparison between the tAUC of VAS after barley and corn cereal consumption.

	Barley (*n* = 15)			Corn (*n* = 12)			
	Baseline	Post 6 Weeks	*p*-Value ^1^ Δ6 Weeks-0 Weeks	Baseline	Post 6 Weeks	*p*-Value ^1^ Δ6 Weeks-0 Weeks	*p*-Value ^1^
tAUC of VAS ^3^							
Hunger	4950 ± 1234 ^2^	3900 ± 2345	0.248	3825 ± 1045	3525 ± 2145	0.972	0.950
Fullness	5475 ± 1892	6300 ± 1789	0.277	6075 ± 1567	5475 ± 1678	0.820	0.020 *
Satisfy	4725 ± 1678	5400 ± 1456	0.095	5175 ± 1367	4575 ± 1567	0.362	0.014 *
Desire to eat	4950 ± 1567	4200 ± 2123	0.285	4275 ± 1989	3975 ± 1890	0.572	0.191

^1^ Analyzed by Wilcoxon signed-rank test (within-group); Mann–Whitney test (between-group). * *p* < 0.05. ^2^ Data are presented as means ± standard deviation. ^3^ VAS, visual analog scale.

## Data Availability

The data presented in this study are available upon request from the corresponding author. The data are not publicly available to protect participant confidentiality.

## Supplementary Materials

The following supporting information can be downloaded at: https://www.mdpi.com/article/10.3390/nu17172801/s1. Figure S1: Individual participant postprandial glucose responses (tAUC) at baseline and after 6 weeks of (a) barley or (b) corn cereal consumption; Figure S2: Postprandial GLP-1 responses over 120 min at baseline and after 6 weeks of barley or corn cereal consumption. S1: CONSORT 2025 checklist item description. Ref. [38] is cited in Supplementary Materials.

## References

[1] World Health Organization (2024). Obesity and Overweight. WHO Fact Sheet. https://www.who.int/news-room/fact-sheets/detail/obesity-and-overweight.

[2] Nam G.E., Kim Y.H., Han K., Jung J.H., Rhee E.J., Lee S.S., Kim D.J., Lee K.W., Lee W.Y. (2020). Obesity fact sheet in Korea, 2019: Prevalence of obesity and abdominal obesity from 2009 to 2018 and social factors. J. Obes. Metab. Syndr..

[3] Korea Disease Control and Prevention Agency Korea National Health and Nutrition Examination Survey (KNHANES). https://knhanes.kdca.go.kr/knhanes/main.do.

[4] Kim B.Y., Kang S.M., Kang J.H., Kang S.Y., Kim K.K., Kim K.B., Kim B., Kim S.J., Kim Y.H., Kim J.H. (2021). 2020 Korean Society for the Study of Obesity guidelines for the management of obesity in Korea. J. Obes. Metab. Syndr..

[5] Kweon S., Kim Y., Jang M.-j., Kim Y., Kim K., Choi S., Chun C., Khang Y.-H., Oh K. (2014). Data resource profile: The Korea national health and nutrition examination survey (KNHANES). Int. J. Epidemiol..

[6] Kim M.-S., Jung B.-M. (2021). A Study on the Dietary and Lifestyle Changes of Middle-Aged Women in the Gwangju Area in the COVID-19 Era. Korean J. Community Nutr..

[7] Bracale R., Vaccaro C.M. (2020). Changes in food choice following restrictive measures due to COVID-19. Nutr. Metab. Cardiovasc. Dis..

[8] Han C., Park S.M., Lim J.-S., Kwon J.-W. (2018). Association of Dietary Factors with Presence and Severity of Septal Deviation: Results of the Korean National Health and Nutrition Survey 2010–2012. Korean J. Otorhinolaryngol. Head Neck Surg..

[9] Song S., Lee J.E., Song W.O., Paik H.-Y., Song Y. (2014). Carbohydrate intake and refined-grain consumption are associated with metabolic syndrome in the Korean adult population. J. Acad. Nutr. Diet..

[10] Zhuo M., Chen Z., Zhong M.-L., Liu Y.-M., Lei F., Qin J.-J., Sun T., Yang C., Chen M.-M., Song X.-H. (2023). The global disease burden attributable to a diet low in fibre in 204 countries and territories from 1990 to 2019. Public Health Nutr..

[11] Kweon S., Yang J., Oh K. (2018). Energy intakes through homemade and dining-out meals in the Korea National Health and Nutrition Examination Survey (KNHANES). Public Health Wkly. Rep..

[12] Reynolds A., Mann J., Cummings J., Winter N., Mete E., Te Morenga L. (2019). Carbohydrate quality and human health: A series of systematic reviews and meta-analyses. Lancet.

[13] Lazaridou A., Biliaderis C.G. (2007). Molecular aspects of cereal β-glucan functionality: Physical properties, technological applications and physiological effects. J. Cereal Sci..

[14] Tosh S.M. (2013). Review of human studies investigating the post-prandial blood-glucose lowering ability of oat and barley food products. Eur. J. Clin. Nutr..

[15] Astbury N.M., Piernas C., Hartmann-Boyce J., Lapworth S., Aveyard P., Jebb S.A. (2019). A systematic review and meta-analysis of the effectiveness of meal replacements for weight loss. Obes. Rev..

[16] Priebe M.G., McMonagle J.R. (2016). Effects of ready-to-eat-cereals on key nutritional and health outcomes: A systematic review. PLoS ONE.

[17] Alminger M., Eklund-Jonsson C. (2008). Whole-grain cereal products based on a high-fibre barley or oat genotype lower post-prandial glucose and insulin responses in healthy humans. Eur. J. Nutr..

[18] Tighe P., Duthie G., Vaughan N., Brittenden J., Simpson W.G., Duthie S., Mutch W., Wahle K., Horgan G., Thies F. (2010). Effect of increased consumption of whole-grain foods on blood pressure and other cardiovascular risk markers in healthy middle-aged persons: A randomized controlled trial. Am. J. Clin. Nutr..

[19] Kohzuma T., Tao X., Koga M. (2021). Glycated albumin as biomarker: Evidence and its outcomes. J. Diabetes Complicat..

[20] Cho Y.-H., Lee S.-y. (2013). Food Intake and Gut Hormones. Korean J. Obes..

[21] Williams D.L., Baskin D.G., Schwartz M.W. (2009). Evidence that Intestinal Glucagon-Like Peptide-1 Plays a Physiological Role in Satiety. Endocrinology.

[22] Matthews D.R., Hosker J.P., Rudenski A.S., Naylor B.A., Treacher D.F., Turner R.C. (1985). Homeostasis model assessment: Insulin resistance and β-cell function from fasting plasma glucose and insulin concentrations in man. Diabetologia.

[23] Flint A., Raben A., Blundell J., Astrup A. (2000). Reproducibility, power and validity of visual analogue scales in assessment of appetite sensations in single test meal studies. Int. J. Obes..

[24] Choi Y.J., Cho J.H., Lee D.H., Song D.J., Kwon Y.J., Baek S.M., Kim Y.J., Jang M.H., Lee D.H., Park H.Y. (2019). Development of Koreans Gut Quotient Measurement Scales. Korean J. Gastroenterol..

[25] Waddell I.S., Orfila C. (2023). Dietary fiber in the prevention of obesity and obesity-related chronic diseases: From epidemiological evidence to potential molecular mechanisms. Crit. Rev. Food Sci. Nutr..

[26] Després J.-P., Lemieux I. (2006). Abdominal obesity and metabolic syndrome. Nature.

[27] Koga M., Kasayama S. (2010). Clinical impact of glycated albumin as another glycemic control marker. Endocr. J..

[28] Kim H., Stote K.S., Behall K.M., Spears K., Vinyard B., Conway J.M. (2009). Glucose and insulin responses to whole grain breakfasts varying in soluble fiber, β-glucan. Eur. J. Nutr..

[29] Tiwari U., Cummins E. (2011). Meta-analysis of the effect of β-glucan intake on blood cholesterol and glucose levels. Nutrition.

[30] Kosmas C.E., Martinez I., Sourlas A., Bouza K.V., Campos F.N., Torres V., Montan P.D., Guzman E. (2018). High-density lipoprotein (HDL) functionality and its relevance to atherosclerotic cardiovascular disease. Drugs Context.

[31] Holst J.J. (2007). The physiology of glucagon-like peptide 1. Physiol. Rev..

[32] Steinert R.E., Feinle-Bisset C., Asarian L., Horowitz M., Beglinger C., Geary N. (2017). Ghrelin, CCK, GLP-1, and PYY(3-36): Secretory controls and physiological roles in eating and glycemia in health, obesity, and after RYGB. Physiol. Rev..

[33] Canfora E.E., Jocken J.W., Blaak E.E. (2015). Short-chain fatty acids in control of body weight and insulin sensitivity. Nat. Rev. Endocrinol..

[34] Zeevi D., Korem T., Zmora N., Israeli D., Rothschild D., Weinberger A., Ben-Yacov O., Lador D., Avnit-Sagi T., Lotan-Pompan M. (2015). Personalized nutrition by prediction of glycemic responses. Cell.

[35] Blaak E.E., Antoine J.M., Benton D., Björck I., Bozzetto L., Brouns F., Diamant M., Dye L., Hulshof T., Holst J.J. (2012). Impact of postprandial glycaemia on health and prevention of disease. Obes. Rev..

[36] Heianza Y., Qi L. (2017). Gene-diet interaction and precision nutrition in obesity. Int. J. Mol. Sci..

[37] Blundell J., De Graaf C., Hulshof T., Jebb S., Livingstone B., Lluch A., Mela D., Salah S., Schuring E., Van Der Knaap H. (2010). Appetite control: Methodological aspects of the evaluation of foods. Obes. Rev..

[38] Hopewell S., Chan A.W., Collins G.S., Hróbjartsson A., Moher D., Schulz K.F., Tunn R., Aggarwal R., Berkwits M., Berlin J.A. (2025). CONSORT 2025 Statement: Updated guideline for reporting randomised trials. BMJ.

