# Supplementary material for: Barley-Based Cereals Enhance Metabolic Health and Satiety in Overweight Korean Adults: A Randomized Trial
Source: Nutrients. 2025 Aug 28;17(17):2801. doi: 10.3390/nu17172801 (PMC12430438; doi:10.3390/nu17172801)
Supplement: Supplementary file 1 [file nutrients-17-02801-s001.zip › supplementary-figure-S1.pdf]

Supplementary Figure S1
Individual participant glucose responses before and after 6-week intervention

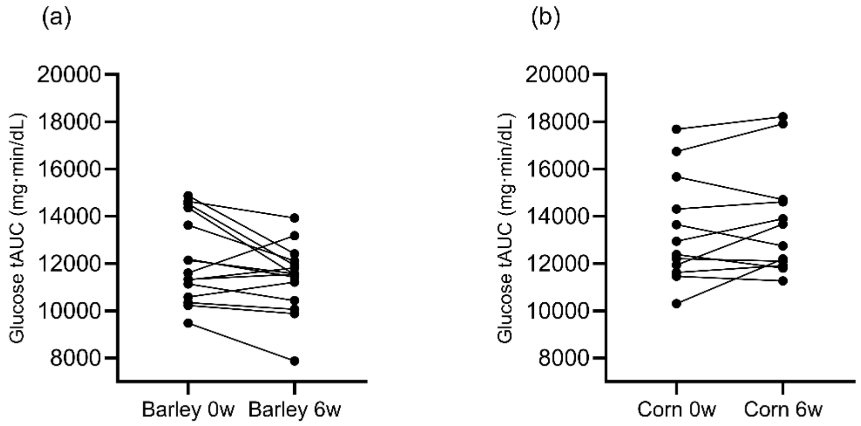

Supplementary Figure S1. Individual participant postprandial glucose responses (tAUC) at baseline and after 6 weeks of (a) barley or (b) corn cereal consumption. Each line represents an individual participant. Abbreviation: tAUC, total area under the curve.
