# Supplementary material for: Barley-Based Cereals Enhance Metabolic Health and Satiety in Overweight Korean Adults: A Randomized Trial
Source: Nutrients. 2025 Aug 28;17(17):2801. doi: 10.3390/nu17172801 (PMC12430438; doi:10.3390/nu17172801)
Supplement: Supplementary file 1 [file nutrients-17-02801-s001.zip › supplementary-figure-S2.pdf]

Supplementary Figure S2

Postprandial GLP-1 responses before and after 6-week intervention

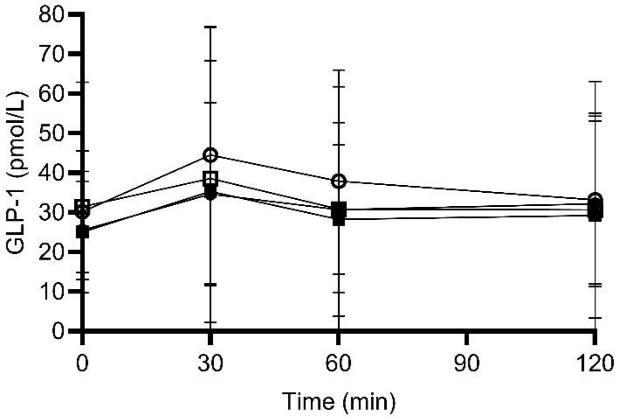

Supplementary Figure S2. Postprandial GLP-1 responses over 120 minutes at baseline and after 6 weeks of barley or corn cereal consumption. ●: Barley baseline; ○: Barley 6 weeks; ■: Corn baseline; □: Corn 6 weeks. Data are presented as means ± SD. Analyzed by Wilcoxon signed-rank test (within-group). * $p < 0.05$ for barley group at 30 min (baseline vs 6 weeks). Abbreviation: GLP-1, glucagon-like peptide-1.
